# Long intergenic non-protein coding RNA 115 (LINC00115) aggravates retinoblastoma progression by targeting microRNA miR-489-3p that downregulates 6-phosphofructo-2-kinase/fructose-2,6-biphosphatase 2 (PFKFB2)

**DOI:** 10.1080/21655979.2022.2037362

**Published:** 2022-02-19

**Authors:** Fang Ji, Chunhua Dai, Meng Xin, Jing Zhang, Yuru Zhang, Shu Liu

**Affiliations:** Department of Ophthalmology, Yantai Affiliated Hospital of Binzhou Medical University, Yantai, China

**Keywords:** RB, LINC00115, miR-489-3p, PFKFB2, miR-489-3p

## Abstract

Long non-coding RNAs (lncRNAs) are key regulators of cancer. However, the role of long intergenic non-protein coding RNA 115 (LINC00115) in the regulation of retinoblastoma (RB) has not yet been studied. The expression levels of LINC00115, microRNA (miR)-489-3p, and 6-phosphofructo-2-kinase/fructose-2,6-biphosphatase 2 (PFKFB2) in RB tissues or cells were detected by quantitative reverse transcription-polymerase chain reaction. The proliferation and migration of cells were detected by the cell counting kit-8 and Transwell assays. Luciferase reporter gene analysis and RNA immunoprecipitation assay were used to validate the target gene interactions predicted by starBase. A xenograft tumor experiment was conducted to validate the in vivo outcomes. The expression levels of LINC00115 and PFKFB2 in RB tissues were higher than those in normal tissues, while miR-489-3p showed the opposite trend. Silencing of LINC00115 inhibited the proliferation and migration of SO-RB50 and HXO-RB44 cells. An inhibitory or facilitated effect on RB tumorigenesis was observed following PFKFB2 silencing or miR-489-3p overexpression, respectively. Moreover, LINC00115 aggravated RB progression by targeting miR-489-3p, which downregulated PFKFB2. This finding improves our understanding of the relationship between LINC00115 and RB. Furthermore, miR-489-3p and PFKFB2 may be used as potential targets for RB prevention and treatment.

## Introduction

Retinoblastoma (RB) occurs in one or both eyes [1], with an incidence of 60% in one eye [2]. It is the most common intraocular malignant tumor in childhood [1], with an incidence of approximately 1/15,000 [2]. There are many treatment methods for RB, including radiotherapy, surgery, and intra-arterial chemotherapy [3], but the main treatment is the complete removal of the affected eyeball; however, the postoperative prognosis remains poor, which is fatal to most children [4]. A large number of studies have shown that mutations in the *RB* gene are the cause of RB [5,6], and that predictive testing before RB is important for controlling the disease progression and subsequent treatment [7], The detection of effective biomarkers involved in tumorigenesis is essential for understanding the molecular mechanism of RB development [8]; however, research on RB pathogenesis is still limited.

Non-coding RNA transcripts, such as long-chain non-coding RNAs (lncRNAs), are critical players in the pathogenesis of cancer, including RB [9]. For example, the overexpression of lncRNA NKILA boosts the proliferative, migratory, and invasive capacities of RB cells [10], LncRNA CANT1 can inhibit the progression of RB by blocking the gene-specific histone methyltransferase supplementation [11]. A decrease in lncRNA PVT1 levels inhibits the expression of Notch2 by upregulating microRNA (miR)-488-3p, thereby inhibiting RB cell proliferation, migration, and invasion, and cell cycle progression, while inducing cell apoptosis [12]. Downregulation of LINC00115 can significantly inhibit the proliferation, migration, and invasion of colorectal cancer cells, while increasing cell apoptosis [13]; LINC00115 promotes metastasis of breast cancer by regulating the expression levels of miR-7 and Kruppel-like factor 4 (KLF4) [14]; LINC00115 can enhance the zinc finger protein 596/enhancer of zeste 2 polycomb repressive complex 2 subunit/signal transducer and activator of transcription 3 signal transduction and promote the growth of glioblastoma [15]; and LINC00115 is involved in the development of bladder cancer [16], human papillomavirus (HPV)-negative cervical cancer [17], lung adenocarcinoma [18], and colon adenocarcinoma [19]; however, the role of LINC00115 in RB has not yet been reported.

miRNAs are small RNAs (18–25 nt) with no translation ability that affect the expression of target genes via the messenger RNA (mRNA) [20]. Dysregulation of miRNAs has been documented in RBs. For instance, low miR-340 expression in RB inhibits cancer cell growth by downregulating the kinesin family member 14 [21]. miR-491-3p interacts with stannin to inhibit tumor metastasis in RB [22]. miR-489-3p is a tumor suppressor that reduces the proliferation, migration, and invasion of bladder cancer cells by targeting histone deacetylase 2 [23]. In addition, miR-489-3p inhibits glioblastoma by targeting the brain-derived neurotrophic factor [24]. Moreover, miR-489-3p affects tumor progression in colorectal cancer [13], renal cell carcinoma [25], and melanoma [26]. These results demonstrate the potential of miR-489-3p as a therapeutic candidate for cancer; however, the underlying mechanisms of miR-489-3p and its target gene in RB have not yet been reported.

Importantly, 6-phosphofructo-2-kinase/fructose-2,6-biphosphatase 2 (PFKFB2) is a member of the PFKFB family, which is a bifunctional family of enzymes that controls the levels of fructose 2, 6-diphosphate [27]. Previous studies have reported the aberrant expression of PFKFB2 in a variety of tumors, including pancreatic cancer [28], hepatocellular carcinoma [29], and gastric cancer [30]. Here, we focused on the effects of PFKFB in RB and investigated the underlying mechanism of LINC00115/miR-489-3p/PFKFB2 axis based on the regulatory network.

In this study, we focused on the function and underlying mechanism of LINC00115 in RB. We hypothesized that LINC00115 promoted the RB cell proliferation, migration, and tumor growth by targeting the miR-489-3p/PFKFB2 axis. Our findings provide a theoretical basis for understanding the pathogenesis of RB, and reveal a promising therapeutic target against RB.

## Materials and methods

### Bioinformatics analysis

starBase algorithm (http://starbase.sysu.edu.cn/) was used to predict the target miRNAs of LINC00115 and target genes of miR-489-3p. At the same time, GSE97508 from the Gene Expression Omnibus Datasets was applied to screen the differentially expressed genes (DEGs) with the following screening criteria: adjusted P < 0.05 and logFC ≥ 1.5. Finally, the screened DEGs were uploaded to the Search Tool for the Retrieval of Interacting Genes/Proteins (STRING) to construct a protein-protein interaction (PPI) network to further identify the key genes in RB.

### Clinical tissues

Samples were obtained from 24 patients with RB. None of the patients had received any systemic or local treatment before tumor tissue resection, and all patients had signed informed consent forms before surgery. This study was approved by the Ethics Committee of Yantai Affiliated Hospital of Binzhou Medical University (Yantai, China). RB and corresponding normal adjacent tissues were stored in liquid nitrogen. All samples were confirmed pathologically and classified according to the International RB Staging System (IRSS) [31].

### Cell maintenance and transfection

Human retinal epithelial cell line (ARPE-19) and human RB cells (Y79, SO-RB50, and HXO-RB44) purchased from ATCC (USA) were cultured in high-glucose Dulbecco’s modified Eagle’s medium containing 15% fetal bovine serum at 37°C with 5% carbon dioxide (CO_2_).

The interference fragment of the LINC00115 gene (GAGTTAGTTTAGTATTCAA(17)) (si-lnc), PFKFB2 gene (si-PFKFB2), or negative control (si-NC) were designed and synthesized by Addgene (Middlesex, UK). In addition, miR-489-3p inhibitor and inhibitor-NC were purchased from SwitchGear Genomics (USA). The reagent used for plasmid transfection was Lipofectamine 2000 (Invitrogen, Carlsbad, CA, USA), and the plasmid incubation solution was opti-MEM (Invitrogen Gibco, USA). When the cell density reached approximately 75%, Lipofectamine 2000, siRNA (50 nM), and inhibitor (75 nM) were introduced into SO-RB50 and HXO-RB44 cells (5 × 10^4^ cells per well, 24-well plate). After 4.5 h of transfection, the medium was replaced with fresh medium, and quantitative reverse transcription-polymerase chain reaction (qRT-PCR) was performed to test the transfection efficiency.

### Transwell assays

SO-RB50 and HXO-RB44 cells (5 × 10^5^ cells/mL) were inoculated into the upper chamber of Transwell inserts (Corning, USA), and serum-free culture medium was added. After 24 h of culture, the cells attached to the lower chamber and those at the bottom of the well were fixed with formaldehyde and washed away. After the fixation solution was stained with crystal violet, the number of migrated cells was counted under a microscope (Olympus. Japan) after washing off the staining solution [32].

### Cell counting kit-8 (CCK-8) assay

Transfected RB cells (1 × 10^5^ cells/mL) were seeded into a 96-well plate for the indicated time and 15 μL of CCK-8 reagent was added to each well until the cell surface was completely covered. After 3 h, the absorbance (450 nm) was measured using a microplate reader (Bio-Tek, USA) [33].

### Nuclear and cytoplasmic separation

The nuclear protein and cytoplasmic protein extraction kit (Biyuntian, China) were used to separate the cytoplasmic and nuclear fractions of SO-RB50 and HXO-RB44 cells following the manufacturer’s protocol. Subsequently, qRT-PCR was used to detect the expression levels of LINC00115 in the cytoplasm and nucleus [34].

### qRT-PCR assay

Total RNA was extracted using an RNA extraction kit (Thermo, USA), and RNA quantity and quality were measured using a NanoDrop microvolume spectrophotometer (ND-1000; NanoDrop Technologies, DE, USA). The SuperScript IV First-Strand Synthesis System (Thermo, USA) was used for cDNA synthesis, followed by qRT-PCR amplification. Real-time PCR assay was performed using the Applied Biosystems StepOne Sequence Detection System (Applied Biosystems, CA, USA) using SYBR green dye (Invitrogen), according to the manufacturer’s instructions. The PCR reaction system consisted of cDNA (10 μL), upstream and downstream primers (10 μM) of the gene (each 2 μL), ddH_2_O (66 μL), and SYBR Green I dye (20 μL). The setting program was: 95°C for 5 min, 94°C for 15s, 55°C for 30s, 72°C for 30s, 4°C for 30 min, 40 cycles. The 2^−ΔΔCt^ method was used to calculate the gene expression [35]. The primers for glyceraldehyde-3-phosphate dehydrogenase (GAPDH), uracil 6 (U6), LINC00115, miR-489-3p, and PFKFB2 were designed and synthesized by Ribobio (Guangzhou, China). The primers used in this study are listed in Table 1.

**Table 1.** The sequences of the primers in this study

Primer	Sequences
LINC00115	Forward: 5’-TGGCTTGTCTTCCATCGTCC-3’
	Reverse: 5’-GCACGAGGGTTGTTACAGGA-3’
PFKFB2	Forward: 5’-AGTCCTACGACTTCTTTCGGC-3’
	Reverse: 5’-TCTCCTCAGTGAGATACGCCT-3’
miR-489-3p	Forward: 5’-GCGCGGTGACATCACATATAC-3’
	Reverse: 5’-AGTGCAGGGTCCGAGGTATT-3’
GAPDH	Forward: 5’-ACCACAGTCCATGCCATCAC-3’
	Reverse: 5’-TCCACCCTGTTGCTGTA −3’
U6	Forward: 5’-AGCCCGCACTCAGAACATC-3’
	Reverse: 5’-GCCACCAAGACAATCATCC-3’
NAME	FROM
LINC00115	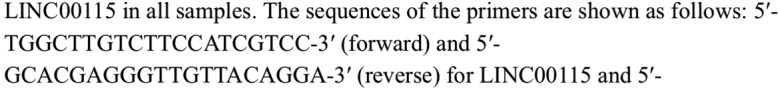
PFKFB2	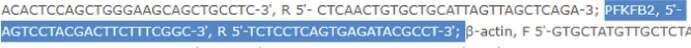
miR-489-3p	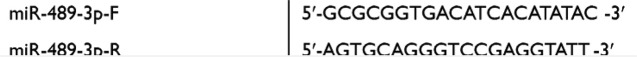
GAPDH	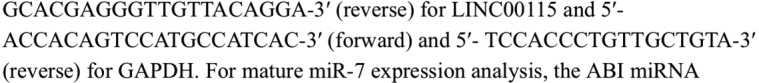
U6	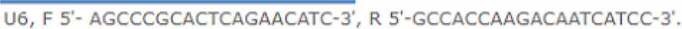

### Dual luciferase reporter gene detection

The predicted wild-type (WT) binding site sequence of LINC00115 or PFKFB2 3’-untranslated region (UTR) and miR-489-3p and the mutant (MUT) were cloned into pmirGLO luciferase reporter vectors. The resultant vectors were pmirGLO-LINC00115-WT, pmirGLO-PFKFB2 3’-UTR-WT, pmirGLO-LINC00115-MUT and pmirGLO- PFKFB2 3’-UTR-MUT. Next, WT and MUT luciferase reporter vectors were co-transfected with miR-489-3p mimic or mimic-NC into SKOV3 and A2780 cells using Lipofectamine 2000. Dual-luciferase reporter assay system (Promega, WI, USA) was used to detect the luciferase activity after 48 h of incubation [32].

### Xenograft model studies

Animal experiments were approved by the Animal Care and Use Committee of the Yantai Affiliated Hospital of Binzhou Medical University (Yantai, China). Control shRNA lentiviral particles (sh-NC) and LINC00115 shRNA lentiviral particles (sh-lnc) (Genepharma, USA) were introduced into SO-RB50 cells (5 × 10^6^), following puromycin selection for 3 weeks. Ten 5-weeks-old BALB/c female nude mice were purchased from the Chinese Academy of Sciences (Shanghai, China). Puromycin-resistant SO-RB50 cells (1 × 10^6^) from sh-NC and sh-lnc groups were subcutaneously injected into 5-weeks-old female BALB/C nude mice (5 mice/group). Tumor size was monitored every 7 d. Five weeks later, the mice were euthanized by administering excessive sodium pentobarbital, and xenograft tumors were excised and weighed [36].

### Western blotting assays

A protein extraction kit (Abcam, USA) was used to extract the proteins from SO-RB50 and HXO-RB44 cells. A bicinchoninic acid assay kit (Beyotime, China) was used to determine the protein concentration. Samples (15 μg) were subjected to constant-voltage electrophoresis at 80 V in 10% sulfate-polyacrylamide gel. Next, the protein was transferred to a polyvinylidene fluoride membrane under a constant current of 200 mA, and the target protein membrane was cut out and sealed with skimmed milk powder at 37°C for 90 min. After blocking, the protein primary antibodies (PFKFB2, cat#bs-5005 R; GAPDH, cat#bs-0755 R; dilution ratio: 1:500; Bioss, China) were incubated with membrane overnight at 4°C. The next day, the protein secondary antibodies corresponding to the primary antibodies were incubated again for 2 h. Finally, the protein bands were visualized using enhanced chemiluminescence (ECL) substrate kit (Amersham Biosciences, Little Chalfont, UK) and analyzed using Image Lab Software (version 4.1; Bio-Rad) [37].

### RNA immunoprecipitation (RIP)

SO-RB50 and HXO-RB44 cells were lysed using the EZMagna Kit (Abcam, USA). The cell extracts were incubated with magnetic beads conjugated with anti-Ago2 (Abcam, USA) or anti-IgG (Abcam, USA) antibodies for 6 h, the magnetic beads were removed after incubation, and RNA was purified and analyzed by qRT-PCR [38].

### Data analysis

All experimental data were obtained from three independently repeated experiments. GraphPad Prism 7.0 (GraphPad Software, La Jolla, CA, USA) was used for all data analysis. All variables in the experiment were expressed as mean ± standard deviation. One-way analysis of variance was used to compare multiple groups, and Student’s t-test was used for comparison between two groups. Statistical significance was set at P < 0.05.

## Results

In this study, we focused on the function and underlying mechanism of LINC00115 in RB. We hypothesized that LINC00115 promotes RB cell proliferation, migration, and tumor growth by targeting the miR-489-3p/PFKFB2 axis. We found that LINC00115 and PFKFB2 in RB were highly expressed, while miR-489-3p showed the opposite trend. Silencing LINC00115 inhibited the proliferation, migration, and tumor growth of RB cells. LINC00115 aggravates RB progression by targeting miR-489-3p, which downregulates PFKFB2. Our findings provide a theoretical basis for further understanding the pathogenesis of RB, suggesting a promising therapeutic target against RB based on LINC00115.

### LINC00115/miR-489-3p/PFKFB2 axis in RB

**Figure 1.**
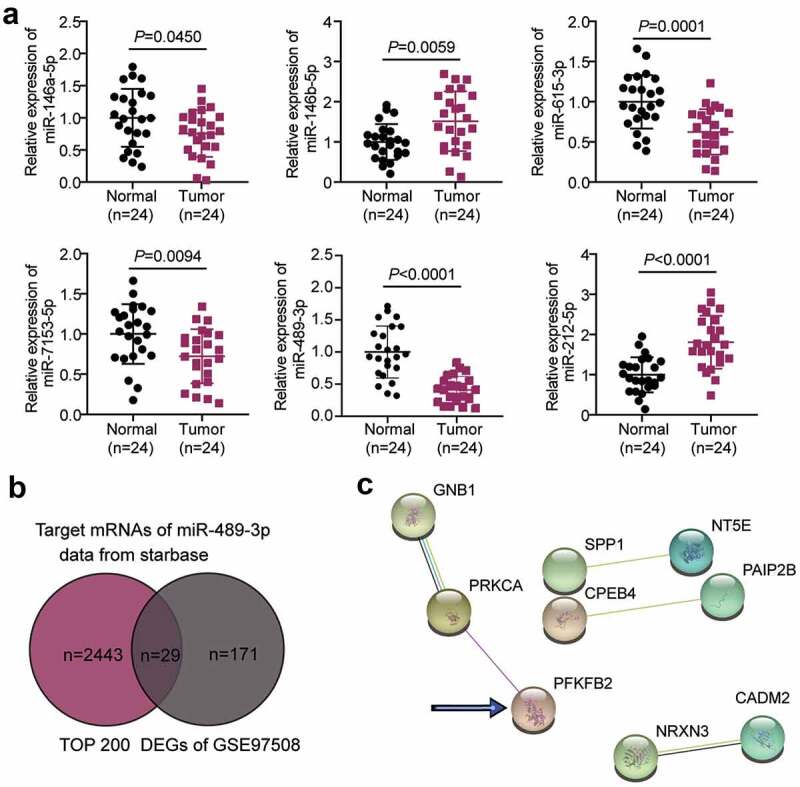
LINC00115/miR-489-3p/PFKFB2 axis in RB. (a) qRT-PCR was used to detect the expression of miR-146a-5p, miR-146b-5p, miR-615-3p, miR-7153-5p, miR-489-3p and miR-212-5p in RB tissues (N = 24) and normal tissues (N = 24). (b) The common/overlapping genes between the target list of miR-489-3p predicted by starbase algorithm and the DEGs in RB from GSE97508. DEGs: differentially expressed genes; DEGs were selected according to adjusted P < 0.05 and logFC≥1.5. (c) The PPI network analysis of the overlapping genes. According to string database, the interaction edges in the PPI network represent the interaction evidence strength. Data are presented as mean ± SD.

LINC00115 has been reported to be an oncogene in various human cancers, including colorectal [13], glioma [15], cervical cancer [17], and breast cancer [14]. However, its role in RB has not yet been studied. According to the starBase algorithm prediction results, there are six target miRNAs of LINC00115: miR-146a-5p, miR-146b-5p, miR-615-3p, miR-7153-5p, miR-489-3p, and miR-212-5p. qRT-PCR was used to detect the expression of these miRNAs in RB and normal tissues. As shown in Figure 1(a), miR-489-3p was most significantly downregulated in cancer tissues. Although miR-489-3p has been reported to be a significant tumor suppressor [23,39–44], its role in RB has not been reported. Hence, miR-489-3p was selected for subsequent experiments. By intersecting the targets of miR-489-3p and the DEGs in RB from GSE97508, 29 common genes were identified (Figure 1(b)). The 29 genes were subsequently uploaded to the STRING database for PPI network analysis. From the PPI network, 9 of the 29 genes demonstrated close relationships (Figure 1(c)). Among the nine genes, PFKFB2 was demonstrated to promote aggression in various cancers [30,45–48] but not RB.

### Interfering with LINC00115 to inhibit the development of RB in vivo and in vitro

**Figure 2.**
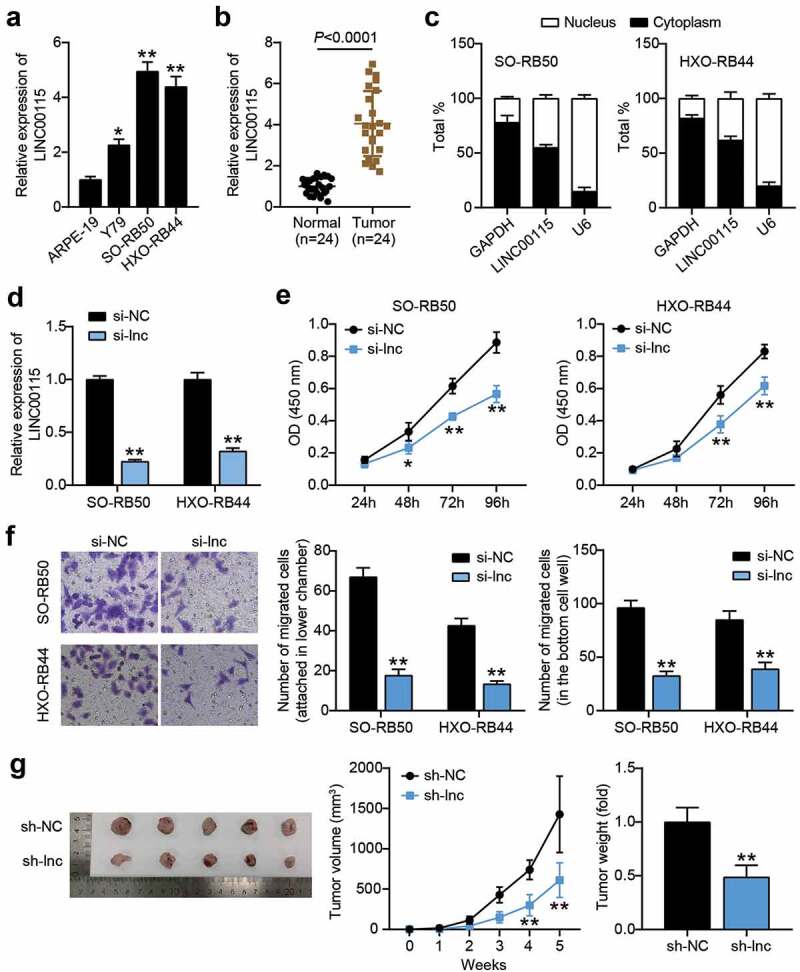
Interference with LINC00115 inhibits the development of RB in vivo and in vitro. (a) qRT-PCR analysis of the expression of LINC00115 in ARPE-19, Y79, SO-RB50, HXO-RB44. *P < 0.05, **P < 0.001, vs. ARPE-19. (b) qRT-PCR was used to detect the expression of LINC00115 in RB tissues (N = 24) and normal tissues (N = 24). (c) Nuclear and cytoplasmic separation to detect the expression of LINC00115 in SO-RB50, HXO-RB44 cytoplasm and nucleus. (d) qRT-PCR was used to detect the expression of LINC00115 in SO-RB50, HXO-RB44 transfected control si-NC or si-lnc. (e) CCK-8 detecting the cell viability of the transfected control si-NC or si-lnc in SO-RB50 and HXO-RB44. (f) Transwell assays detectingcell migration in SO-RB50 and HXO-RB44 transfected with control si-NC or si-lnc. **P < 0.001, vs. si-NC. (g) Representative images of tumors in nude mice 4 weeks after injection of si-NC or si-lnc SO-RB50 cells. **P < 0.001, vs. sh-NC. Data are presented as mean ± SD. N = 3, repetition = 3.

Since the differential expression of LINC00115 has been reported in different cancers, we also tested the expression of LINC00115 in RB cell lines and tumor tissues. The results are shown in Figure 2(a). LINC00115 expression levels of RB50 and HXO-RB44 were higher than those of normal cells ARPE-19. Considering the high expression of LINC00115, these two RB cells were used for subsequent assays. LINC00115 expression in RB tissues was also higher than that in adjacent normal tissues (Figure 2(b)), suggesting a correlation between LINC00115 and RB development. To investigate the clinical significance of LINC00115 in RB, all patients with RB were divided into two groups: the high-expression group and the low-expression group. We then analyzed the relationship between LINC00115 expression and the clinicopathological features of RB patients and found that the high expression of LINC00115 was significantly associated with choroidal invasion, optic nerve invasion, and TNM stage (Table 2). However, there were no statistical correlations with age, gender, tumoral laterality, differentiation, and tumor size (Table 2). Nuclear and cytoplasmic separation showed that LINC0115 was located in the cytoplasm of SO-RB50 and HXO-RB44 cells (Figure 2(c)), implying an interaction between lncRNAs and miRNAs. To further verify the specific relationship between LINC00115 and the development of RB, an interference fragment (si-lnc) of LINC00115 was designed and synthesized. The data showed that LINC00115 expression in SO-RB50 and HXO-RB44 was inhibited in the si-lnc group (P < 0.01), indicating that the interference was effective (Figure 2(d)). The CCK-8 assay showed that the proliferation of RB cells was reduced when LINC00115 was silenced (Figure 2(e)). Migration experiments showed that after interfering with the expression of LINC00115, the number of migrations of SO-RB50 and HXO-RB44 decreased (P < 0.01, figure 2(f)), indicating that LINC00115 silencing mitigates the migration capacity of RB cells. At the same time, in vivo experiments showed that the tumor growth of SO-RB50 cells treated with sh-LINC00115 was slower after subcutaneously inoculated, which showed that the weight and volume of tumors injected with interference fragments were smaller than those of the sh-NC group (P < 0.01, Figure 2(g)); The above results proved that the interference LINC00115 inhibits the growth of RB in vivo and in vitro.

**Table 2.** Correalations between FEZF1-AS1 and clinicopathological features in retinoblastoma

Features	*N = 24*	LINC00115 expression		*P*
		High (n = 12)	Low (n = 12)	
Age(years)				0.400
≤ 4	15	6	9	
> 4	9	6	3	
Gender				0.680
Male	14	6	8	
Female	10	6	4	
Choroidal invasion				0.009*
No	15	4	11	
Yes	9	8	1	
Optic nerve invasion				0.027*
No	16	5	11	
Yes	8	7	1	
Tumor laterality				0.640
Unilateral	18	10	8	
Bilateral	6	2	4	
Differentiation				0.371
Well/moderately	7	5	2	
Poorly	17	7	10	
TNM stage				0.027*
I–II	16	15	11	
III–IV	8	7	1	
Tumor size (mm)				0.100
≤ 10	13	4	9	
> 10	11	8	3	

Notes: The difference between high- and low-LINC00115 expression groups was determined by Fisher test; *P < 0.05.

### LINC00115 targets miR-489-3p in RB cells

**Figure 3.**
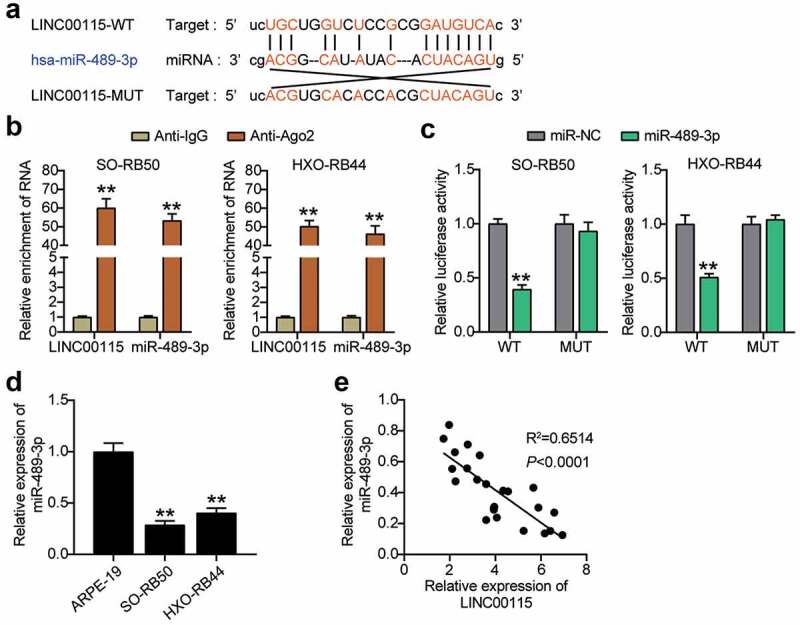
LINC00115 targets miR-489-3p in RB cells. (a) Starbase V2.0 online database predicts the target molecule of LINC00115. (b) RNA immunoprecipitation detects the interaction of LINC00115 and miR-489-3p with Ago2. **P < 0.001, vs. IgG. (c) The effect of miR-489-3p mimic for dual luciferase reporter gene detection on the luciferase activity of LINC00115 wild type and mutant type. **P < 0.001, vs. miR-NC. (d) qRT-PCR detects the expression of miR-489-3p in ARPE19, SO-RB50 and HXO-RB44. **P < 0.001, vs. ARPE-19. (e) The correlation between the expression of miR-489-3p and LINC00115 in RB tissues was evaluated by Pearson analysis. Data are presented as mean ± SD. N = 3, repetition = 3.

To further prove the specific mechanism by which LINC00115 affects the development of RB, the Starbase V2.0 online database predicted that LINC00115 could target miR-489-3p in RB cell lines (Figure 3(a)). RIP analysis showed that LINC00115 and miR-489-3p interacted with Ago2 in the microribonucleoprotein complex, rather than IgG, in SO-RB50 and HXO-RB44 cells (Figure 3(b)), suggesting that LINC00115 and miR-489-3p interact directly. The binding interaction between miR-489-3p and LINC00115 was validated by a luciferase reporter assay. The data showed that miR-489-3p mimic co-transfected with wild-type LINC00115 reduced the luciferase activity of RB cells, but co-transfection with mutant LINC00115 did not (Figure 3(c)). Moreover, the expression of miR-489-3p in RB cell lines and tissues was measured, and the results showed that miR-489-3p levels in SO-RB50 and HXO-RB44 cells were lower than those in ARPE-19 cells (Figure 3(d)). At the same time, Pearson correlation analysis was performed on the expression trends of LINC00115 and miR-489-3p in RB tissues. The results showed that the expression of LINC00115 and miR-489-3p was negatively correlated (Figure 3(e)). Collectively, LINC00115 and miR-489-3p have an interaction relationship in RB, and LINC00115 can target miR-489-3p.

### miR-489-3p inhibitor reverses LINC00115 interference-induced inhibition of

#### RB progression in vitro

**Figure 4.**
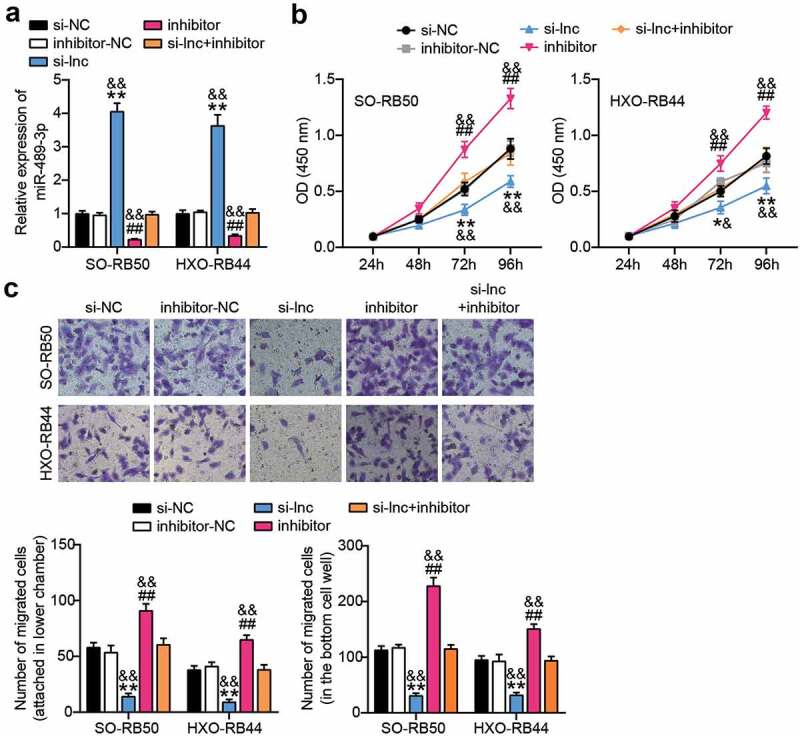
miR-489-3p inhibitors can reverse the inhibitory effect of interfering with LINC00115 on RB. (a) qRT-qPCR analysis detecting the effect of INC00115 in SO-RB50 and HXO-RB44 on the expression of miR-489-3p. (b) CCK-8 assays detecting the effect of the interaction between LINC00115 and miR-489-3p in SO-RB50 and HXO-RB44 on cell proliferation. (c) Transwell assays detecting the effect of the interaction between LINC00115 and miR-489-3p in SO-RB50 and HXO-RB44 on cell migration. *P < 0.05, **P < 0.001, vs. si-NC; ##P < 0.001, vs. inhibitor-NC; & P < 0.05, &&P < 0.001, vs. si-lnc+inhibitor. Data are presented as mean ± SD. N = 3, repetition = 3.

To further explore the relationship between LINC00115 and miR-489-3p, miR-489-3p inhibitors were used to study the physiological activities of SO-RB50 and HXO-RB44 cells. qRT-PCR showed that interference with the expression of LINC00115 activated the expression of miR-489-3p in RB cells (P < 0.01), and the expression of miR-489-3p activated upon LINC00115 silencing in RB cells was neutralized by miR-489-3p inhibitor (P < 0.01, Figure 4(a)), suggesting that si-LINC00115 restored the expression of miR-489-3p in RB. On this basis, the effects of LINC00115 and miR-489-3 on the proliferation and migration abilities of both RB cells. As shown in Figure 4(b), the inhibitor of miR-489-3p reversed the inhibitory effect of interfering with LINC00115 on cell proliferation. Likewise, the inhibitor of miR-489-3p reversed the inhibitory effect of interfering with LINC00115 on cell migration (Figure 4(c)). These data indicate that the miR-489-3p inhibitor reverses the inhibitory effect of interfering with LINC00115 gene-induced thyroid cancer in vitro.

#### miR-489-3p targets PFKFB2 in RB cells

**Figure 5.**
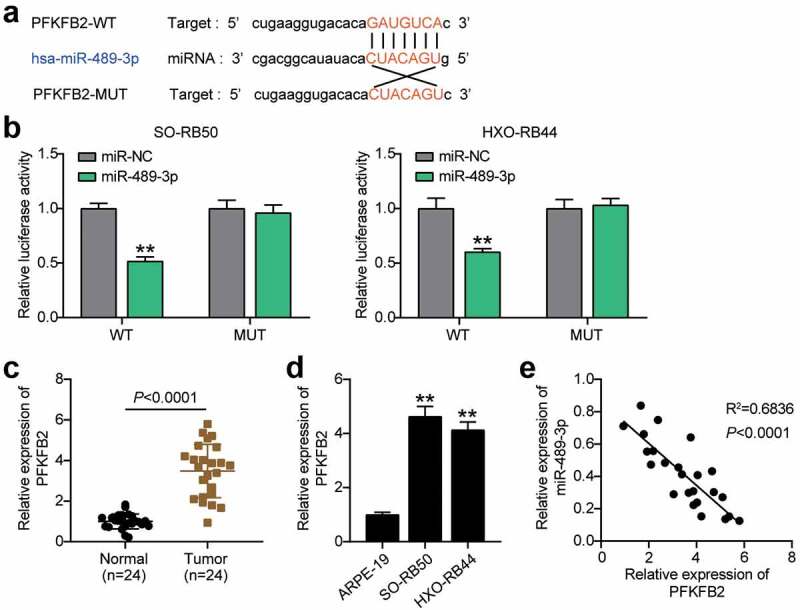
miR-489-3p targets PFKFB2 in RB cells. (a) Starbase V2.0 online database predicting the target molecule of miR-489-3p. (b) The effect of miR-489-3p mimic for dual luciferase reporter gene detection on the luciferase activity of wild-type and mutant PFKFB2. **P < 0.001, vs. miR-NC. (c) qRT-PCR was used to detect the expression of PFKFB2 in RB tissues (N = 24) and normal tissues (N = 24). (d) qRT-PCR detecting the expression of PFKFB2 in ARPE-19, SO-RB50 and HXO-RB44. **P < 0.001, vs. ARPE-19. (e) The correlation between the expression of miR-489-3p and PFKFB2 in RB tissues was evaluated by Pearson analysis. Data are presented as mean ± SD. N = 3, repetition = 3.

Further bioinformatics analysis showed that miR-489-3p could target PFKFB2 (Figure 5(a)). In addition, miR-489-3p mimics reduced the luciferase activity of wild-type PFKFB2, but did not affect the luciferase activity of mutant PFKFB2 (Figure 5(b)), suggesting that miR-489-3p and PFKFB2 could interact with each other. In addition, qRT-PCR showed that the expression of PFKFB2 in RB tissues and cells was higher than that in normal tissues (Figure 5(c-d)). Interestingly, the expression of miR-489-3p and PFKFB2 in RB tissues was negatively correlated (p < 0.01, Figure 5(e)). In summary, miR-489-3p can regulate each other with PFKFB2 in RB cells, and PFKFB2 is involved in tumor progression.

#### miR-489-3p affects the progression of RB by regulating the expression of PFKFB2

**Figure 6.**
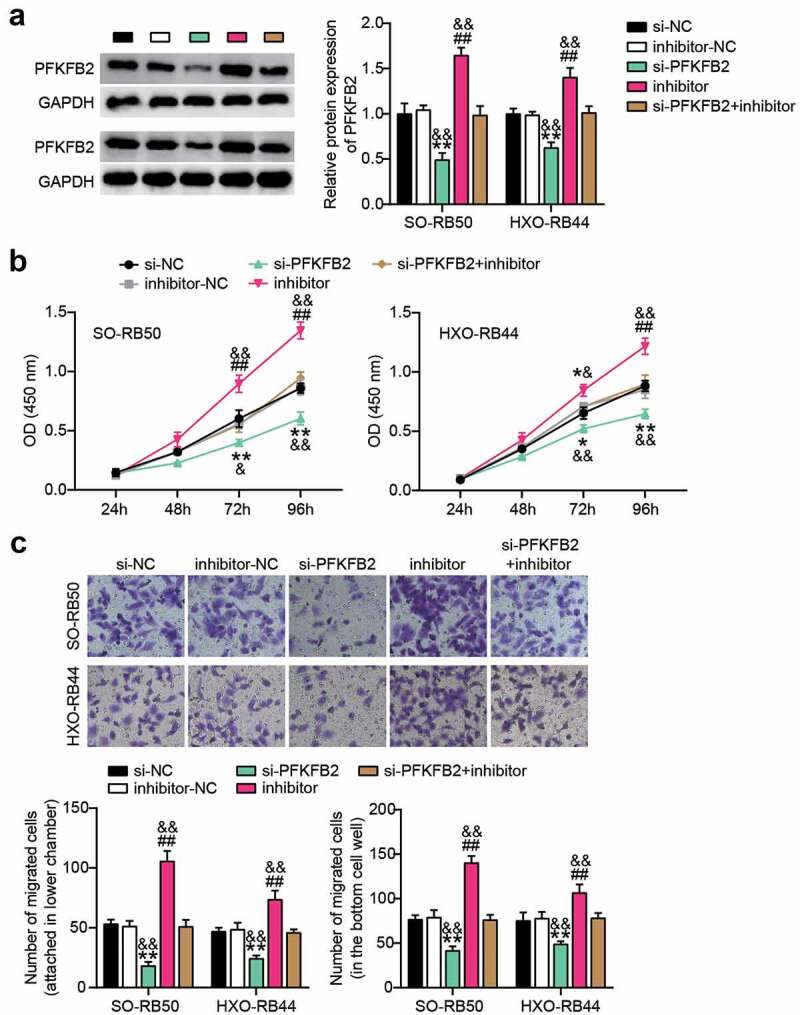
Interference with the expression of miR-489-3p can weaken the control of tumor development by inhibiting PFKFB2. (a) Western blotting detecting the effect of miR-489-3p on the expression of PFKFB2 in SO-RB50 and HXO-RB44 cells. (b) CCK-8 assays detecting the effect of the interaction of miR-489-3p on PFKFB2 in SO-RB50 and HXO-RB44 on cell proliferation. (c) Transwell assays detecting the effect of the interaction of miR-489-3p on PFKFB2 in SO-RB50 and HXO-RB44 on cell migration. *P < 0.05, **P < 0.001, vs. Si-NC; ##P < 0.001, vs. inhibitor-NC; &P < 0.05, &&P < 0.001, vs. si-PFKFB2+ inhibitor. Data are presented as mean ± SD. N = 3, repetition = 3.

Western blotting analysis showed that PFKFB2 protein expression was decreased after interfering with PFKFB2 expression, but with the addition of miR-489-3p inhibitor, PFKFB2 protein expression levels were increased in RB cells (Figure 6(a)). Moreover, PFKFB2 interference and miR-489-3p inhibitor co-treatment of RB cells restored the PFKFB2 expression. At the same time, CCK-8 and Transwell assays showed that silencing PFKFB2 inhibited the proliferation and migration of RB cells, while the inhibition of miR-489-3p weakened the inhibitory effects of PFKFB2 on tumor cell proliferation and migration (Figure 6(b-c)).

## Discussion

Here, we found abnormal overexpression of LINC00115 in RB tissues and cell lines, and determined that LINC00115 silencing suppressed RB cell proliferation and migration in vitro and inhibited tumor growth in vitro. At the clinical level, we found that high expression of LINC00115 predicted worse pathology in patients with RB. Mechanistically, interaction of LINC00115 with miR-489-3p upregulated PFKFB2 expression, ultimately inhibiting RB progression.

The mechanism of action of lncRNA LINC00115 in a variety of diseases his known [13,14]. LINC00115 promotes breast cancer metastasis by regulating the expression levels of miR-7 and KLF4 [14]; LINC00115 is significantly upregulated in HPV-negative cervical cancer cells and can promote the proliferation, migration, and migration of these cells [17]; however, its role in RB remains unknown. Herein, we found increased expression levels of LINC00115 in RB tissues and cells, and interference with LINC00115 expression inhibited the cell viability, migration ability, and tumor size in RB. This new finding is consistent with the role of LINC00115 in other cancers. Furthermore, high expression of LINC00115 was found to be associated with poor histopathological phenotypes in RB. These data suggest that LINC00115 plays a carcinogenic role in RB and may be used as a therapeutic target for RB at the clinical level.

The effects of miR-489-3p on different types of tumors have been reported. miR-489-3p acts as an anti-oncogenic miRNA by attenuating the expression of distal-less homeobox 1, which shows pro-tumor effects [40]; miR-489-3p can inhibit the proliferation, migration, and migration of melanoma cells by inhibiting SIX1 [26]. Therefore, miR-489-3p inhibits cancer progression. Similar to previous reports, in this study, we found that miR-489-3p plays an anti-tumor role in RB. Specifically, miR-489-3p levels were downregulated in RB tissues and cell lines, and its low expression inhibited RB cell proliferation and migration.

LncRNAs can affect disease progression by co-regulating the expression of their downstream target genes via cooperative/spongy miRNAs, which form the competitive endogenous RNA (ceRNA) regulatory networks [49]. In recent years, increasing ceRNAs have been shown to closely regulate the occurrence and development of RB. For example, metastasis-associated lung adenocarcinoma transcript 1 acts as a ceRNA to inhibit the expression of miR-655-3p, ultimately regulating the ATPase family AAA domain containing 2 and promotes the oncogenic phenotype [50]. Five prime-to-Xist promotes RB progression by targeting the miR-320a/with-no-lysine kinase 1 axis [51]. Therefore, understanding ceRNA crosstalk will expand our understanding of gene regulatory networks and aid in the development of novel therapeutic strategies and approaches for RB. In our study, we found that LINC00115 could target miR-489-3p in RB cells, and miR-489-3p could inhibit the luciferase reporter gene activity of LINC00115. Moreover, rescue analysis showed that silencing miR-489-3p reversed the inhibitory effects of low LINC00115 expression on RB cell proliferation and migration. These findings reveal a new role of miR-489-3p in RB and confirm the sponge effect of LINC00115 on miR-489-3p.

Tumor cells need to increase glucose metabolism through glycolysis and other pathways to meet the energy requirements of cell proliferation. The 6-phosphofructose-2-kinase/fructose-2,6-bisphosphatase (PFKFB1-4) family is the key to glucose metabolism modulators [28]. Studies have reported that PFKFB2 plays a role in regulating the development of acute kidney injury [52];, and PFKFB2 can be targeted by miR-1297 to inhibit the proliferation of osteosarcoma [45]. Many studies have shown that miR-489-3p and PFKFB2 play a role in a variety of diseases [23,29]. In our study, miR-489-3p targeted PFKFB2. In addition, PFKFB2 was highly expressed in RB tissues and cells, and inhibition of miR-489-3p can upregulate the expression of PFKFB2, and cell viability and migration ability were also upregulated. These data suggest that PFKFB2 can be used as a target for the development of RB, suggesting that PFKFB2 is targeted by miR-489-3p to promote the malignant behavior of RB.

This research needs to be further improved. In this study, only the prediction and verification of molecular targets was carried out, while specific signal pathways were not identified; therefore, these pathways need to be studied further via furture in vivo experiments.

## Conclusion

Based on the above results, it can be concluded that LINC00115 predicts a poor pathological phenotype and acts as an oncogenic factor in RB. Moreover, LINC00115 controls RB progression by targeting miR-489-3p to downregulate the expression of PFKFB2. This discovery can provide new insights for the study of RB, and LINC00115 may serve as a potential target for the clinical diagnosis and treatment of RB.

## Data Availability

The datasets used and/or analyzed during the current study are available from the corresponding author on reasonable request.

## Highlights

- LINC00115 and PFKFB2 levels in RB tissues and cells were higher than those in normal tissues, while miR-489-3p showed an opposite trend.
- Silencing of LINC00115 inhibited the proliferation, migration, and tumor growth of RB cells.
- An inhibitory or facilitated effect on RB tumorigenesis was observed following PFKFB2 silencing or miR-489-3p overexpression, respectively.
- LINC00115 sponged miR-489-3p, and miR-489-3p targeted PFKFB2 in RB.
